# Metainflammation in Diabetic Coronary Artery Disease: Emerging Role of Innate and Adaptive Immune Responses

**DOI:** 10.1155/2016/6264149

**Published:** 2016-08-16

**Authors:** Vivekanandhan Aravindhan, Haridoss Madhumitha

**Affiliations:** ^1^Department of Genetics, Dr. ALM. PG. IBMS, University of Madras, Chennai 600113, India; ^2^AU-KBC Research Centre, MIT Campus of Anna University, Chennai 600044, India

## Abstract

Globally, noncommunicable chronic diseases such as Type-2 Diabetes Mellitus (T2DM) and Coronary Artery Disease (CAD) are posing a major threat to the world. T2DM is known to potentiate CAD which had led to the coining of a new clinical entity named diabetic CAD (DM-CAD), leading to excessive morbidity and mortality. The synergistic interaction between these two comorbidities is through sterile inflammation which is now being addressed as metabolic inflammation or metainflammation, which plays a pivotal role during both early and late stages of T2DM and also serves as a link between T2DM and CAD. This review summarises the current concepts on the role played by both innate and adaptive immune responses in setting up metainflammation in DM-CAD. More specifically, the role played by innate pattern recognition receptors (PRRs) like Toll-like receptors (TLRs), NOD1-like receptors (NLRs), Rig-1-like receptors (RLRs), and C-type lectin like receptors (CLRs) and metabolic endotoxemia in fuelling metainflammation in DM-CAD would be discussed. Further, the role played by adaptive immune cells (Th1, Th2, Th17, and Th9 cells) in fuelling metainflammation in DM-CAD will also be discussed.

## 1. Introduction

In recent years, noncommunicable chronic diseases such as Type-2 Diabetes Mellitus (T2DM) and Coronary Artery Disease (CAD) are posing a major threat to the world irrespective of geographical and ethnic boundaries [1]. T2DM is known to potentiate CAD which had led to the coining of a new clinical entity named diabetic CAD (DM-CAD), leading to excessive morbidity and mortality [1]. The synergistic interaction between these two comorbidities is through sterile inflammation which is now being addressed as metabolic inflammation or metainflammation [2]. Metainflammation is due to the dysfunction of the immune system which acts like a double edged sword: at optimal level it confers protection against pathogens; at the suboptimal level it leads to immunodeficiency; at supraoptimal level it leads to inflammation. The pathogenesis of DM-CAD is complex with the involvement of multiple factors including genetic predisposition and various environmental factors like high fat diet, sedentary life style, and chronic stress [1]. Though the association of inflammation with T2DM and CAD was envisioned as early as in 1800s the mechanisms mediating these inflammatory responses were not clearly known [3]. T2DM arises due to insulin resistance (IR) during early stages, which in turn arises due to the inflammation of the insulin target organs (adipose, skeletal muscle, and liver) [4]. IR leads to increased insulin demand and thereby causes rapid exhaustion of pancreatic beta cells due to overproduction, eventually leading to insulin deficiency (ID) [4]. Thus, late stage of T2DM is characterized by combined ID and IR leading to hyperglycemia, eventually leading to endothelial dysfunction [5].

CAD is a macrovascular complication characterized by enhanced extravasation and accumulation of inflamed macrophages under the tunica intima, wherein they engulf the oxidized lipids and become foam cells, leading to the formation of atherosclerotic plaques (atherogenesis) [5]. There are 4 important mechanisms that majorly contribute to the development of hyperglycemia induced cardiovascular damage: (1) increased sorbitol production due to activation of polyol pathway, (2) increased O-GlcNAcylation of cytosolic proteins, (3) activation of protein kinase C, and (4) increased formation of Advanced Glycation End-Product (AGE) [6]. The underlying common element in all these mechanisms is the increased production of reactive oxygen species (ROS) in endothelial cells under diabetic condition [6]. Recently redox stress has also been linked to neoangiogenesis as seen in microvascular complications (HIF-1*α* activation) and metainflammation (NF-*κ*B activation) [6]. DM induced hyperglycemia accelerates the process of atherosclerosis, with greater infiltration of inflammatory macrophages and T lymphocytes and increased inflammation of the coronary artery [7]. Metainflammation augments atherogenesis by directly promoting arterial lipid deposition and inducing the proliferation and migration of smooth muscle cells [7]. It also indirectly promotes atherogenesis by augmenting other risk factors of CAD including dyslipidemia, diabetes, and hypertension [6, 7]. Thus there are several immune factors involved in atherosclerosis which involve cells (endothelial cells, macrophages, and lymphocytes), cytokines, chemokines, acute phase proteins, and adhesion molecules [8–10]. Among these C-reactive protein (CRP), Interleukin-6 (IL-6), and Tumour Necrosis Factor (TNF-*α*) have been used as predictive markers of CAD as evidenced by various epidemiological studies [10]. Thus, metainflammation plays a pivotal role during both early and late stages of T2DM and also serves as a link between T2DM and CAD. However, the exact mechanism behind the initiation of inflammation as seen in these two conditions is not clearly known. In this review, a summary of the role played by innate and adaptive immune responses in setting up metainflammation in DM-CAD would be presented.

## 2. Role of Innate Metainflammation in DM-CAD

**Figure 1:**
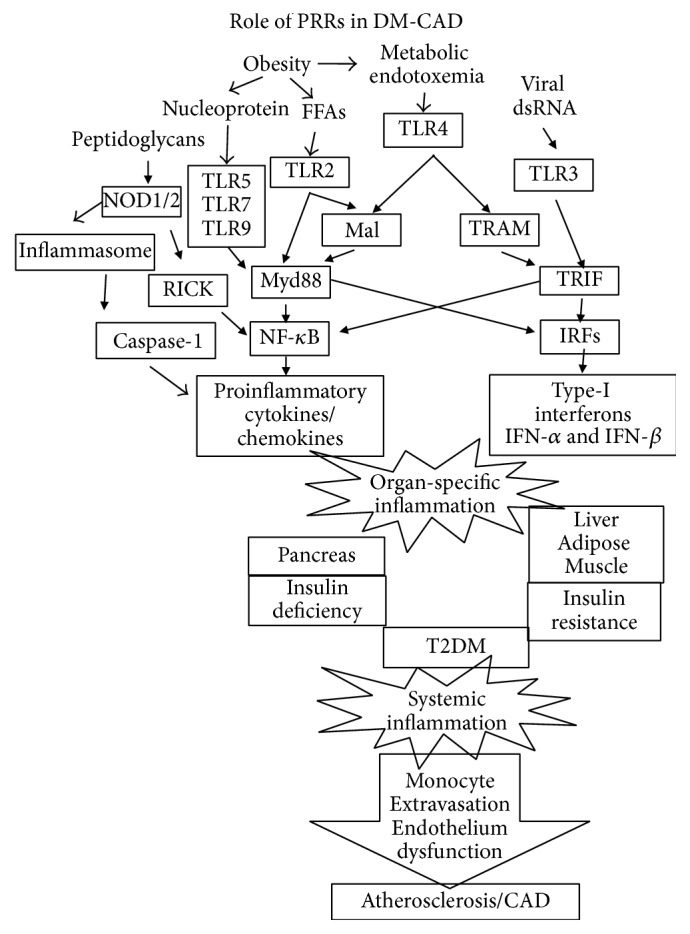
Role of innate immune response in triggering metainflammation associated with DM-CAD. Pattern recognition receptors (PRRs) are the well characterized innate immune receptors which trigger metainflammation following recognition of both pathogen associated molecular patterns (PAMPs) and damage associated molecular patterns (DAMPs). Viral nucleic acids, endotoxins, and peptidoglycans are some of the PAMPS which are released into the circulation following metabolic endotoxemia. Free fatty acids and self-nucleoproteins are some of the endogenous ligands which act as DAMPs. The end result is the activation of NF-*κ*B and IRFs which in turn activate proinflammatory cytokines and type-1 interferons, respectively. These inflammatory mediators destroy pancreatic beta cells leading to insulin deficiency and induce inflammation at insulin target organs leading to insulin resistance (IR) eventually precipitating in Type-2 Diabetes. Long standing diabetes induces systemic inflammation leading to monocyte activation and endothelial dysfunction leading to the extravasation of monocytes and formation of atherosclerotic plaques.

The innate immune system serves as a first-line defense mechanism against invading pathogens. Unfortunately, the same system also serves as the first-line initiator of metainflammation in DM-CAD. The pattern recognition receptors (PRRs) which include Toll-like receptors (TLRs), NOD-like receptors (NLRs), Rig-1-like receptors (RLRs), and C-type lectin like receptors (CLRs) serve as the major arsenal of innate immunity in detecting unique pathogen associated molecular patterns (PAMPs) and thereby alerting the immune system [11] (Figure 1). However, apart from these well characterized receptors, new members are being added to this ever increasing list. These receptors are widely distributed in immune and nonimmune cells to enable rapid detection of pathogens and immediate activation of the immune system (danger-signal hypothesis), resulting in immunity. In fact, these receptors act as bridges between the innate and adaptive arms of the immune responses [12]. However, under certain pathogenic conditions, the same receptors, which detect the pathogens, also detect commensals and self-molecules and activate the immune system resulting in metainflammation. Metainflammation in DM-CAD is characterized by increased serum levels of proinflammatory cytokines like TNF-*α*, IL-6, and IL-1*β* [13, 14] and anti-inflammatory cytokines like IL-10 and Transforming Growth Factor-beta (TGF-*β*) [15]. DM associated hyperglycemia and hyperlipidemia might also fuel metainflammation. Nonenzymatic glycation of proteins and oxidised lipids can bind to innate immune receptors and can activate them. Inappropriate activation of these receptors in various organs is believed to be a major contributory factor towards the increased secretion of these cytokines. Apart from this, increased ROS production could also be an underlying cause for the increased proinflammatory cytokine secretion through enhanced NF-*κ*B activation [16].

## 3. Role of Toll-Like Receptors (TLRs) in DM-CAD

Among the various PRRs, the TLRs were the earliest and thus the most well characterized group of receptors. TLRs were first identified in* Drosophila*, wherein they were found to confer immunity against fungal infection [17]. Later, homologs of these receptors were identified in the human genome and were found to perform similar functions. Till date, at least 10 members of TLR family have been identified and characterized in humans and have been implicated in a wide range of inflammatory conditions including cancer, infection, autoimmunity, immunodeficiency, and graft rejection [18]. However, recent studies in animals and humans have shown their involvement in metabolic diseases especially DM-CAD [19–21]. These receptors apart from recognizing the PAMPs, present in the pathogens, also detect damage associated molecular patterns (DAMPs), present in the host culminating in inflammation [22]. Immune cells like B cells, monocytes, and dendritic cells predominantly express TLRs compared to NK cells and T cells which show lesser expression. Activation of TLRs results in the increased secretion of proinflammatory cytokines such as TNF-*α* and IL-6 which are known to induce IR leading to T2DM [23] and promote atherogenesis leading to CAD [24] (Figure 1). Increased expression of TLR4 has been reported in the adipose tissue [25], fatty liver [26], and skeletal muscle [27] of both mice and humans. However studies carried out in our lab have shown strong downregulation of TLR2 and TLR4 in B cells and monocytes of newly diagnosed T2DM subjects which was largely due to the upregulation of immunomodulatory enzymes indoleamine-2,3-dioxygenase (IDO), arginase-1, and heme oxygenase-1, indicating that chronic hyperglycemia can impair immunity by downregulating TLR expression [28]. This opens up a susceptibility window where newly diagnosed subjects are under increased risk to infections [28]. Enhanced expression of TLR1, TLR2, and TLR4 in atherosclerotic plaques has been reported in humans [29]. During high fat diet, these receptors get activated which results in the inhibition of insulin signaling augmenting atherogenesis [30]. TLR1, TLR2, TLR4, and TLR6 which are abundantly expressed in monocytes cooperate with CD14, CD36 (scavenger receptor), and complement receptors in transforming these monocytes into foam cells [31].

Activation of TLR results in the activation of NF-*κ*B which has been identified as a “master regulator” of inflammation [32]. NF-*κ*B activation occurs either through Mal:MyD88 pathway (which is sometimes referred to as MyD88-dependent pathway) or through TRAM:TRIF pathway (which is sometimes referred to as MyD88-independent pathway) [32]. Activated NF-*κ*B induces proinflammatory cytokines which when secreted reinforce the action of TLRs, setting up a positive feedback loop [32]. Interestingly, apart from NF-kB, activation of IRF3 (by TLR3 and TLR4) and IRF7 (by TLR7, TLR8, and TLR9) results in the secretion of type-1 interferons (interferon-*α* and interferon-*β*) [33]. These interferons, like the proinflammatory cytokines, act in an autocrine fashion reinforcing the TLR stimulation, via IFN-*αβ*R-STAT1 pathway [33]. Apart from cytokines and interferons, TLR stimulation leads to the secretion of variety of chemokines which are largely under the control of NF-*κ*B and STAT-1 regulation [34]. While TLR induced secretion of proinflammatory cytokines, type-1 interferons, and chemokines promotes inflammation, TLR induced secretion of anti-inflammatory cytokine IL-10 (and in some cases TGF-*β*) is the major self-limiting pathway involved in curtailing inflammation [35]. IL-10, through the JAK1-STAT3 pathway, negatively regulates TLR signaling by degrading IRAK4 and TRAF6 thereby dampening MyD88-dependent pathway (but not the MyD88-independent pathway) [36]. Apart from NF-kB, IRFs, and STAT1, AP-1 and ATF3 are the other major transcription factors involved in TLR signaling and their role in DM-CAD is yet to be deciphered [32]. While the role of proinflammatory cytokines in promoting IR and atherogenesis is well known, the involvement of type-1 interferons, chemokines, and anti-inflammatory cytokines in these pathogenic processes is less well studied.

## 4. Role of NOD-Like Receptors (NLRs) in DM-CAD

NOD-like receptors (NLRs) are the second group of pattern recognition receptors (PRRs) which are important components of the host innate immune responses that regulate metainflammation. Though NLR and TLR pathways are mediated through different adaptors, they induce the expression of proinflammatory cytokines by activating NF-*κ*B signaling [37]. Various cell types express NOD1 and NOD2 including epithelial cells, dendritic cells [38], keratinocytes [39], macrophages [40], and the Paneth cells [41]. Among the NLR family members, NOD1 and NOD2 recognize bacterial peptidoglycans resulting in the activation of MAPK and NF-*κ*B signaling leading to transcriptional upregulation of proinflammatory cytokines [41]. Along with NALP3 they promote the assembly of large multiprotein complexes called “inflammasomes” [42]. These inflammasomes in turn activate the proteolytic caspase-1 which cleaves and activates the proinflammatory cytokines IL-1*β* and IL-18 that signal cell damage [42]. Further, like TLRs they are also capable of activating type-1 interferons, via IRF3 [43]. Since NLRs and TLRs act in a similar fashion in provoking the inflammatory response, NLRs could also play a complimentary role in the pathogenesis of DM-CAD [44] (Figure 1). NOD proteins mediated metainflammation and IR has been demonstrated in many cell types [45–47]. Monocytes from T2DM subjects have shown upregulation of NOD1 and NOD2 mRNA which also correlated with HOMA-IR, indicating its role in T2DM [48]. NOD1 mRNA was markedly upregulated in the adipose tissue of diet-induced (DIO), but not genetically susceptible (ob/ob), obese mice [45]. Stimulation of NOD1 with a synthetic ligand Tri-DAP induced proinflammatory chemokines (MCP-1, RANTES, and MIP-2) and cytokines (TNF-*α* and IL-6) in 3T3-L1 adipocytes [45]. A similar proinflammatory profile was also observed in human primary adipocytes stimulated with NOD1 which suppressed insulin signaling [45]. Like NOD1, activation of NOD2 in L6-myotubes induced IR within 3 h, which was characterized by a reduction in insulin-stimulated glucose uptake, GLUT4 translocation, and IRS-1 and Akt-1 phosphorylation [46]. These results showed that NOD2 alone is capable of acutely inducing metainflammation and IR in muscle cells [46]. NOD1/2 KO mice were protected from high fat diet-induced metainflammation, lipid accumulation, and IR [49]. Conversely, direct activation of NOD1 in wild type mice induced IR within 6 h [49]. Oral administration of NOD1 ligand elicited minor changes in systemic inflammation yet caused pronounced IR in adipose tissue, muscle, and liver [49]. Not limited to T2DM, the role of NLRs is also signified in CAD [50]. Bacterial peptidoglycans (PG), the natural ligands of NLRs, were observed in human atherosclerotic plaques and were associated with plaque vulnerability [51]. Oral administration of NOD1 ligands into mice induced vascular inflammation leading to coronary arteritis [52]. Activation of NOD1 in a murine model induced cardiac dysfunction and modulated cardiac fibrosis and cardiomyocyte apoptosis and other pathological processes involved in CAD [53]. Thus, both NOD1 and NOD2 play a pronounced role in setting up metainflammation in DM-CAD.

## 5. Role of RLRs in DM-CAD 

RLRs which belong to RNA helicases family have three members, namely, (1) Retinoic acid-Inducible Gene-I (RIG-I), (2) Melanoma Differentiation Associated 5 (MDA5), and (3) Laboratory of Genetics and Physiology 2 (LGP2). These receptors specifically recognize viral RNA and activate the immune system [11]. Upon activation, RIG-I and MDA5 are recruited to the IPS-1 adaptor which is localized on the outer mitochondrial membrane. IPS-1, via TRAF3-TANK-NAP1 complex, recruits TBK1-IKK*ε*-DDX3 complex and activates IRF3 and IRF7 simultaneously. IPS-1 also recruits TRADD and forms a complex with FADD-RIP-1-TRAF6 activating NF-*κ*B, via IKK. Activated IRFs and NF-*κ*B in turn activate type-1 interferons and proinflammatory cytokines, respectively. In an IPS-1 independent manner, RLRs promote inflammasome assembly and processing of pro-IL-1*β* and pro-IL-18 cytokines [11]. Like TLRs, RIG-1 also contributes to *β*-cell dysfunction indicating its role in metabolic regulation [54]. Under conditions of metabolic surplus, RIG-1 induces the blocking of Src/STAT3 signaling thereby arresting the beta cells from entering into G1 phase [54]. LGP2, the third member of this family, acts as a negative regulator of RIG-1 and MDM5 and inhibits inflammation [55]. Whether LGP2 induced negative regulation of RIG-1 and MDM5 is beneficial to T2DM and CAD is not yet known.

## 6. Role of CLRs in DM-CAD 

C-type lectin like receptors (CLRs) are Ca^2+^ dependent glycan-binding proteins that share a unique carbohydrate-recognition domain (CRD) [56]. It includes Type-1 (DEC205 and Macrophage Mannose Receptor (MMR)) and Type-2 (Dectin-1, Dectin-2, Mincle, DC-SIGN, and DNGR-1) membrane proteins and a soluble receptor (Mannose Binding Lectin (MBL)) [57]. Generally, CLRs recognize complex carbohydrates which decorate bacterial and fungal cell wall and activate the immune system [57]. However, under pathogenic conditions like T2DM, it is highly probable that the same receptors can recognize modified host glycans and inappropriately activate the immune system resulting in metainflammation. The modified host glycans which can bind to these receptors and activate them are yet to be characterized. Upon activation, CLRs like Dectin-1 and DC-SIGN which have ITAM signaling domain can directly activate signaling by recruiting downstream effectors, while those like Dectin-2 and Mincle which lack the ITAM signaling domain associate with other receptors like Fc*γ*R and Fc*ε*R and augment their signaling capacity [57]. Unlike TLRs, NLRs, and RLRs, CLRs do not activate IRFs and induce type-1 interferon secretion. They activate proinflammatory cytokine secretion, via NF-*κ*B, AP-1, and NF-AT [57]. It has been reported that Mincle is induced in M1 macrophages in the adipose tissue under obesity condition thereby suggesting a role in obesity-induced inflammation [58]. The role of other CLRs in metainflammation as seen in DM-CAD is yet to be deciphered.

## 7. Metabolic Endotoxemia in DM-CAD

Even though PRR stimulation has now been identified as a major event in setting up the metainflammation (as seen in DM-CAD), the exact trigger for PRR stimulation remains largely unknown since these receptors can be triggered by both endogenous (PAMPs) and exogenous (DAMPs) ligands. Recently, metabolic endotoxemia has emerged as a major culprit in activating PRRs and setting up metainflammation. Increased gut permeability due to changes in gut microbiota has recently been described in both T2DM and CAD [59]. Because of the leaky gut effect, increased efflux of LPS into systemic circulation takes place which, in turn, is detected by the PRRs [60]. But, more than the actual endotoxin levels, the levels of endogenous anti-endotoxin antibodies (EndoCAb), LPS binding protein (LBP), and soluble CD14 (sCD14) were found to be more important in determining the activity of endotoxin [61]. These three components play an important role in conditions of acute inflammation like septicemia. Even though metabolic endotoxemia was previously reported in both T2DM and CAD [62, 63], in T2DM it is associated with significantly reduced levels of EndoCAb, with no apparent change in the levels of sCD14 and LBP (unpublished data) while in CAD it is associated with significantly elevated levels of EndoCAb and decreased levels of sCD14 with no change in LBP levels [61]. Thus, depending on the relative levels of these accessory proteins, the endotoxin can bind to different PPRs and can initiate different types of metainflammation. While engagement of TLRs predominantly promotes IL-6 and TNF-*α* secretion [32], engagement of NLRs is known to activate inflammasomes resulting in the enhanced processing and secretion of IL-1*β* and IL-18 [44]. Apart from the secretion of proinflammatory cytokines, both TLRs and NLRs are known to induce the secretion of anti-inflammatory cytokines such as IL-10 and TGF-*β*, which counteracts the effect of proinflammatory cytokines and maintain immune homeostasis [32, 44]. An imbalance between the activities of pro- and anti-inflammatory cytokines disrupts the immune homeostasis and would pave the way for metainflammation as seen in DM-CAD.

## 8. Role of Adaptive Metainflammation in DM-CAD

**Figure 2:**
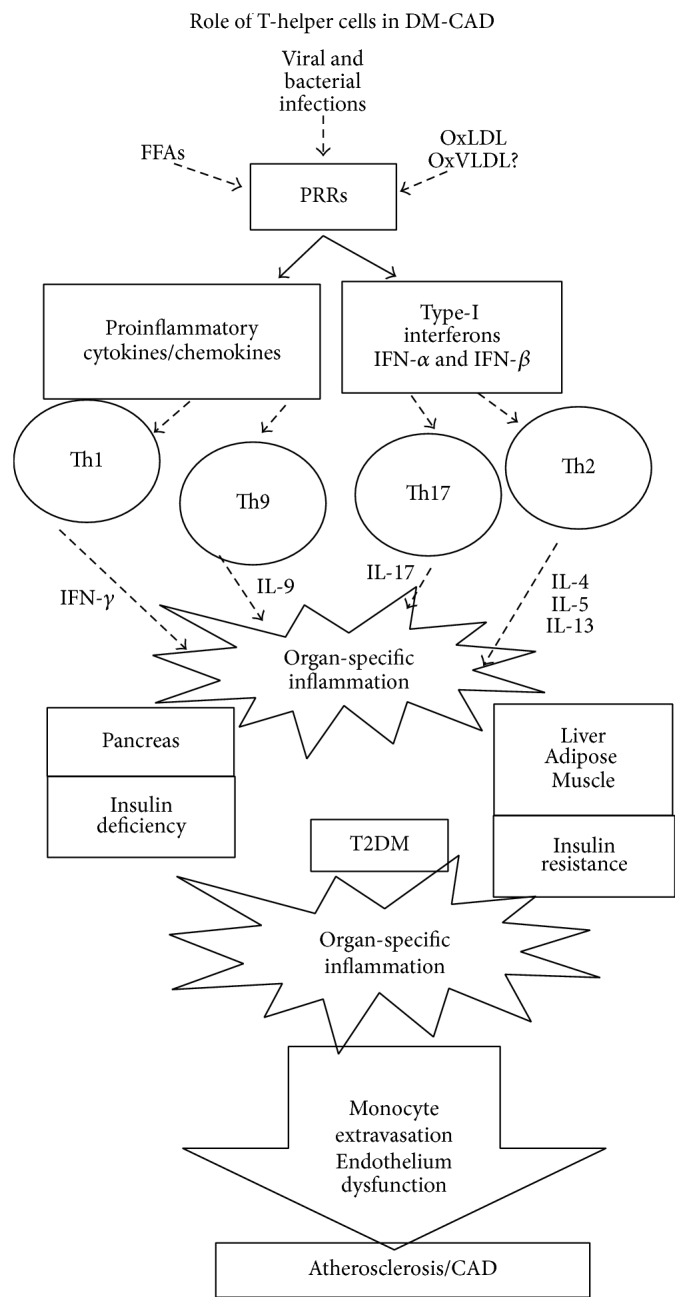
Role of adaptive immune response in triggering metainflammation associated with DM-CAD. CD4^+^T-helper cells are the most well characterized work horses of the adaptive immune system which trigger metainflammation. Inflammation triggered by PRRs is translated to T cells by the APC-T cell interaction which results in the recruitment of these activated cells into pancreases, adipose, liver, and skeletal muscle reinforcing the metainflammation set by PRRs. Depending upon the relative proportions of these cell types pancreatic beta cell apoptosis and IR in insulin target tissues can get aggravated precipitating in Type-2 Diabetes. Long standing diabetes induces systemic inflammation leading to monocyte activation and endothelial dysfunction leading to the extravasation of monocytes and formation of atherosclerotic plaques.

The presence of activated T cells in human adipose tissue (in the case of T2DM) and in atherosclerotic plaque (in the case of CAD) has been identified several years ago indicating the involvement of adaptive immunity in these disease conditions [64] (Figure 2). Adaptive immune cytokines include both Th polarizing and T cell effector cytokines which together shape the adaptive arm of the immune response. While the former is largely secreted by professional antigen presenting cells (APCs) and acts on naïve T cells, the latter is predominantly secreted by polarized T cells and acts on other immune/nonimmune cells. Earlier reports, including ours, have shown significantly increased levels of proinflammatory cytokines like TNF-*α*, IL-6, IL-1*β*, and GM-CSF in T2DM and CAD [14, 15, 65–67]. However, reports documenting the levels of adaptive immune cytokines in DM-CAD are scant. Recently, several novel T cell cytokines like IL-33 [68], IL-17 [69], and IL-9 [70] have been described. While IL-12 has long been known as the master regulator of Th1 polarization, IL-33 has recently emerged as a master regulator for Th2 polarization [71]. Upon activation, T lymphocytes differentiate into T-helper 1 (Th1) and Th2 subsets secreting either Th1 (Interferon- (IFN-) *γ* and IL-2) or Th2 cytokines (IL-4, IL-5, and IL-13), respectively [72]. Th17 and Th9 cell types are newly discovered Th subtypes which secrete IL-17 and IL-9 and play an important role in neutrophil recruitment and mucosal immunity, respectively [72].

## 9. Th1 Cytokines in DM-CAD 

In general, the association of Th1 cytokines with adipose inflammation is well documented in both animals and humans. Pacifico et al. showed increased frequency of Th1 cells in obese children [73]. Wegner et al. showed increased levels of serum IL-12 in T2DM subjects that was associated with IR [74]. Our studies on serum cytokine profiling on subjects with metabolic syndrome indicated strong positive correlation of both IL-12 and IFN-*γ* levels with fasting blood sugar, triglycerides, HOMA-IR, and hsCRP and strong negative correlation with adiponectin [75]. In the murine diet-induced obesity model, Kintscher et al. showed early recruitment of Th1 cells into adipose tissue that precedes even macrophage infiltration and IR [76]. However, the exact mechanism by which IFN-*γ*, the signature cytokine of Th1 cells, brings about IR is not clearly known. IFN-*γ* might exert its action by interfering with the insulin signaling and insulin-stimulated glucose uptake, which might eventually lead to IR and T2DM [77]. Apart from their role in IR, Th1 cells also play a critical role in the initiation, progression, and rupture of atherosclerotic plaque leading to CAD [78]. Jonasson et al. have reported that most of the cells in the atherosclerotic plaque express HLA-DR, indicating continuous activation by IFN-*γ* [79]. The same group has demonstrated the expression of IL-2 and IFN-*γ* in a large proportion of the plaque cells [80]. In apoE KO mice, IFN-*γ* was shown to potentiate atherosclerosis through both local and systemic effects [81]. IFN-*γ* has also been proposed as a component of five panel markers for the prediction of CAD in symptomatic patients referred for coronary angiography [82]. Our studies on serum cytokine profiling in DM-CAD subjects indicated strong Th1 polarization during transition from T2DM/CAD to DM-CAD signifying the importance of Th1 polarization in the disease process [83]. Thus, in the light of the available literature, it seems that Th1 cytokines might worsen IR and promote atherogenesis in DM-CAD.

## 10. Th2 Cytokines in DM-CAD

Compared to Th1 cytokines, the role played by Th2 cytokines in IR and atherogenesis is still an enigma. In a recent study, decreased serum levels of IL-13 in T2DM subjects were reported which was implicated in impaired glucose uptake and metabolism [84]. Chang et al. have demonstrated the role of IL-4 in improving insulin sensitivity and glucose tolerance in an animal model of diet-induced obesity [85]. Winer et al. showed that adoptive transfer of Th2 cells in Rag1 KO, diet-induced obese mice reversed weight gain and IR [86]. Recently, we have reported decreased levels of serum IL-33 in T2DM subjects, while the other Th2 cytokines like IL-4 and IL-13 were significantly increased [87]. In contrast to Th1 cells, Th2 cells are rarely detected within the atherosclerotic lesions [88]. In line with these reports, we found enhanced Th1 cytokine profile in CAD subjects with significant decrease in Th2 cytokine levels [83]. Accumulating evidence suggests that an imbalance in the Th1/Th2 cytokines with enhanced Th1 immune response and suppressed Th2 response has an important role in the transition of T2DM/CAD to DM-CAD [83, 89]. Thus, in the light of the available literature the increased levels of Th2 cytokines in T2DM implicate a countermeasure to inhibit Th1 immunity and thereby IR. However, when this system fails we see enhanced Th1 polarization eventually precipitating in atherogenesis and CAD.

## 11. Th17 Cytokines in DM-CAD

IL-17 cytokine is produced by T-helper cell subset called Th17 which has been widely associated with autoinflammatory (irritable bowel syndrome) and autoimmune (rheumatoid arthritis and multiple sclerosis) [90] diseases. Th17 polarization is mediated through IL-1*β*, IL-6, TNF-*α*, and TGF-*β* and is stabilized by IL-21 and IL-23 [90]. Like other T cell cytokines, IL-17 might also play a role in metabolic diseases like obesity, dyslipidemia, IR, hypertension, and cardiovascular diseases which remains largely unexplored [90]. High glucose was shown to drive the expression of IL-17 in Jurkat T cells implicating the involvement of Th17 cells in T2DM [91]. However, unlike Th1 cytokines, the reports on IL-17 levels in T2DM are highly contradictory. Previously, increased levels of IL-17 were reported in T2DM [92]. However, our studies on subjects with diabetic nephropathy have indicated decreased levels of IL-17 under diabetic conditions [93] with no major difference in IL-23 levels (unpublished data). Even in diabetic retinopathy the serum levels of IL-17 were significantly reduced compared to control group [94]. Since the synthesis of IL-17 is influenced by other cytokines such as TGF-*β* which induces its secretion at lower concentrations and inhibits its secretion at higher concentrations [90], more mechanistic studies are needed to decipher the mechanism behind reduced IL-17 levels in T2DM. IL-17 might also have a pathogenic role in CAD. Studies carried out on CAD-prone apoE KO mice showed significantly elevated levels of plasma IL-17 and infiltration of IL-17 producing Th17 cells into the atherosclerotic plaques [95]. Further, neutralization of IL-17 with a soluble form of IL-17A receptor significantly reduced the size and number of atherosclerotic lesions [95]. In humans, apart from IL-17, IL-21 and IL-23 were also detected in the atherosclerotic plaques and were strongly associated with venerability of plaque rapture [96]. Thus, in the light of the available literature it seems that while IL-17 might offer some protection against IR in DM, it might worsen atherogenesis in DM-CAD.

## 12. Th9 Cytokines in DM-CAD

Th9 cells are recently discovered Th cell subset which undergoes polarization in the presence of IL-4 and TGF-*β* [97]. IL-9 exhibits proinflammatory activity in the experimental models of inflammation [97]. Its role in allergies has been well demonstrated [97]. But studies examining its association with metabolic disorders are limited and are contradictory [98]. Previously, significantly increased levels of plasma IL-9 were reported in T2DM subjects [98]. However, our results showed significantly reduced levels of IL-9 in T2DM subjects which correlated positively with renal parameters [93]. In CAD-prone apoE KO mice, IL-9 exerts proatherosclerotic effects by inducing VCAM-1 expression and thereby promoting macrophage infiltration and atherosclerotic plaque formation [99]. In humans, increased levels of IL-9 in atherosclerotic disorders were seen both systemically and within the lesion, suggesting a role for the IL-9/IL-9R axis in the atherosclerotic process, potentially involving IL-17 mediated mechanisms [100]. Thus, in the light of the available literature it seems that while IL-9 might offer some protection against IR, it worsens atherogenesis in DM-CAD.

## 13. Conclusion

Metabolism and immunity are essential requirements for survival. Mounting an effective immune response requires major changes to metabolic pathways. Similarly, immune mediators (such as cytokines) also dictate changes in metabolism making the communication bidirectional [101]. Thus, the fast emerging field of immunometabolism underpins the pathogenesis of metabolic diseases like T2DM and CAD [101]. Understanding the immune-metabolic interface is daunting. One contemporary issue is characterizing the sources and mediators of metainflammation, which was first characterized in adipose tissue but is now known to be present in many different tissues including liver, muscle, and arteries. Although, the metainflammation is hypothesized to arise from chronic nutrient excess, the sensors which detect these signals are only now beginning to emerge. PRRs have now emerged as major sensors which sense nutrient excess and fuel metainflammation. Once the signal is sensed, it is then transferred to Th cells by means of APC-T cell communication fuelling metainflammation. Interestingly, changes in metabolism also affect the immune response completing the loop [101]. As can be seen in this review even though metainflammation seems to be a common denominator for T2DM and CAD distinct qualitative and quantitative differences were noted between the two conditions indicating that not all metainflammations are the same and each metabolic disease is characterized by a unique inflammatory profile with distinct cytokines and inflammatory cells. However, caution should be noted in interpreting these data since most of these studies are cross-sectional and hence the cause-effect relationship cannot be determined. The apparent disparities noted on serum cytokine profiling between populations could be either due to ethnic differences or due to small sample sizes used or due to heterogeneity of the disease condition. Only parallel studies conducted on different ethnic populations with large sample sizes can solve this issue. Compared to serum cytokine profiling, studies looking at immune cells actually secreting these cytokines are limited and hence future studies should envision identifying the immune cells secreting these cytokine and not just look at serum cytokine levels. Finally, these studies would gain translational value only when the therapeutic utility of monoclonal antibodies against these cytokines is tested in animal models and clinical trials.

## Abbreviations

PRRs: Pattern recognition receptors
PAMPs: Pathogen associated molecular patterns
DAMPs: Damage associated molecular patterns
LPS: Lipopolysaccharide
TLR: Toll-like receptors
NLR: NOD-like receptors
RLR: Rig-1-like receptors
CLR: C-type lectin like receptors
NOD1: Nucleotide-binding oligomerization domain-containing protein 1
RIG-1: Retinoic acid-inducible gene 1
LBP: LPS binding protein
sCD14: Soluble cluster of differentiation 14
EndoCAb: Endotoxin core antibody
IL: Interleukin
TNF: Tumour Necrosis Factor
TGF: Tumour growth factor
Th: T-helper
IR: Insulin resistance
CAD: Coronary Artery Disease
T2DM: Type-2 Diabetes Mellitus
NF-*κ*B: Nuclear factor kappa-light-chain-enhancer of activated B cells
IRF: Interferon regulatory factor
STAT: Signal transducer and activator of transcription
AP-1: Activating protein 1.
